# Case Report: Dasatinib-associated chylothorax and occult *Bartonella henselae* infection in pediatric leukemia: a case report and systematic review

**DOI:** 10.3389/fphar.2026.1897328

**Published:** 2026-09-28

**Authors:** Caili Liu, Ziting Xia, Runxin Lu, Hongye Cai, Chunxi Fu, Shuai Peng, Liang Huang, Xiaoxi Lu

**Affiliations:** 1 Dujiangyan People’s Hospital, Dujiangyan City, Sichuan, China; 2 Department of Pediatric Hematology and Oncology, West China Second University Hospital, Sichuan University, Chengdu, China; 3 Key Laboratory of Birth Defects and Related Diseases of Women and Children, Sichuan University, Ministry of Education, Chengdu, China; 4 Department of Pharmacy/Evidence-Based Pharmacy Centre, West China Second University Hospital, Sichuan University, Chengdu, China; 5 Children’s Medicine Key Laboratory of Sichuan Province, Chengdu, China; 6 NMPA Key Laboratory for Technical Research on Drug Products In Vitro and In Vivo Correlation, Chengdu, China

**Keywords:** Bartonella henselae, chylothorax, dasatinib, drug safety, pediatric leukemia

## Abstract

Dasatinib is a second-generation tyrosine kinase inhibitor (TKI) used in Philadelphia chromosome-positive (Ph+) acute lymphoblastic leukemia (ALL), and chylothorax is one of its rare but serious lymphatic complications. We encountered a 14-year-old boy with Ph + acute lymphoblastic leukemia (ALL) who developed bilateral chylothorax while on dasatinib, with concurrent *B. henselae* infection (cat-scratch disease) confirmed in the pleural fluid. Prompted by this index case, we performed a systematic review of the literature to explore the potential association between dasatinib-associated chylothorax and *Bartonella henselae* infection. We searched multiple databases up to March 2026 and identified 24 cases of TKI-associated chylothorax and 11 cases of leukemia-related cat-scratch disease. In our index case, the pleural fluid triglyceride concentration reached 20.40 mmol/L (≈1806 mg/dL)—the highest value reported to date—and mNGS detected *B. henselae* together with other organisms. After dasatinib was discontinued and the patient received chest drainage, somatostatin, fasting, and anti-infective therapy with doxycycline plus rifampicin, imaging showed near-complete resolution of the effusion, and repeat mNGS confirmed clearance of *B. henselae*. A formal causality assessment using the Naranjo scale gave a score of 7, indicating a “probable” adverse drug reaction. This case illustrates that lymphatic and infectious complications can coincide in the same patient. Dasatinib-mediated inhibition of Src family kinases may simultaneously impair lymphatic endothelial integrity and suppress T-cell-mediated immune surveillance, thereby facilitating both chylous leakage and opportunistic infection. Clinicians managing paediatric patients receiving long-term dasatinib therapy should remain vigilant for both lymphatic complications and atypical opportunistic infections.

## 1 Introduction

Dasatinib is a second-generation tyrosine kinase inhibitor (TKI) that hits multiple targets, including BCR-ABL, Src family kinases, and platelet-derived growth factor receptor β (PDGFR-β) (Li et al., 2010). It has become a cornerstone of treatment for Philadelphia chromosome-positive (Ph+) acute lymphoblastic leukemia (ALL) and chronic myeloid leukemia (CML), offering better event-free and overall survival than imatinib (Abou Dalle et al., 2024; Shen et al., 2020). That said, it comes with a range of adverse effects—rash, gastrointestinal upset, myelosuppression, and fluid retention are all common, and pleural effusion shows up in about 15%–35% of treated patients (Kronick et al., 2023). Chylothorax, where chyle accumulates in the pleural space, is a much rarer but potentially life-threatening lymphatic complication. So far, fewer than 30 cases have been reported worldwide, and paediatric data are especially scarce (Al-Abcha et al., 2019; Alqattan et al., 2022; Bajaj et al., 2023; Baloch et al., 2017; Bradt et al., 2022; Chen et al., 2020; Ferreiro et al., 2016; Ga et al., 2023; Hickman et al., 2020; Hsu et al., 2021; Huang et al., 2015; Justin et al., 2025; Kelly et al., 2022; Liu et al., 2023; Makimoto et al., 2022; Martínez et al., 2023; McGonegal and Baskar, 2024; Molina et al., 2020; Nasi et al., 2022; Ng et al., 2024; Pai and Huang, 2025; Paul et al., 2021; Sasaki et al., 2019; Yang et al., 2016).

Cat-scratch disease, caused by *B. henselae*, is usually a self-limited infection after feline contact. In immunocompromised patients, however—particularly those with haematologic malignancies—the infection can become disseminated or present in atypical ways even without a clear scratch or bite, which makes the diagnosis tricky (Jabbour and Kantarjian, 2025; Petrova et al., 2004). What has never been documented, though, is the combination of dasatinib-induced chylothorax and a concurrent occult *Bartonella henselae* infection occurring in the same patient.

To date, concurrent dasatinib-associated chylothorax and occult *B. henselae* infection have not been systematically described. Herein, we report a pediatric patient with Ph + ALL who developed severe bilateral chylothorax during long-term dasatinib therapy accompanied by pleural-fluid-confirmed *B. henselae* infection. We additionally performed a systematic review of published cases to characterize the clinical manifestations, pharmacological mechanisms, diagnostic strategies, and management approaches associated with these rare complications.

## 2 Methods

This study was conducted in accordance with the PRISMA 2020 statement. PubMed, CNKI, VIP, and Wanfang databases were searched from database inception to March 2026 using combinations of the following keywords: “dasatinib,” “tyrosine kinase inhibitor,” “chylothorax,” “*B. henselae*,” “cat-scratch disease,” and “leukemia.” Only human studies published in English or Chinese were included.

Two independent reviewers screened titles, abstracts, and full-text articles. Eligible studies included case reports or case series describing TKI-associated chylothorax or leukemia-associated *B. henselae* infection. Narrative reviews, experimental studies, and reports lacking sufficient clinical details were excluded.

Data extracted included patient demographics, underlying disease, TKI exposure duration, pleural fluid characteristics, treatment strategies, microbiological findings, and clinical outcomes. Due to the descriptive nature of this study, protocol registration was not performed.

## 3 Results

### 3.1 Study selection

The search identified 1,192 records in total. After 86 duplicates were removed, 1,106 titles and abstracts were screened, and 45 full-text articles were evaluated for eligibility. In the end, 35 case reports met the inclusion criteria—24 describing TKI-associated chylothorax and 11 describing *B. henselae* infection in leukemia. One of these publications reported three cases. The PRISMA flow diagram is provided in Figure 1.

**FIGURE 1:**
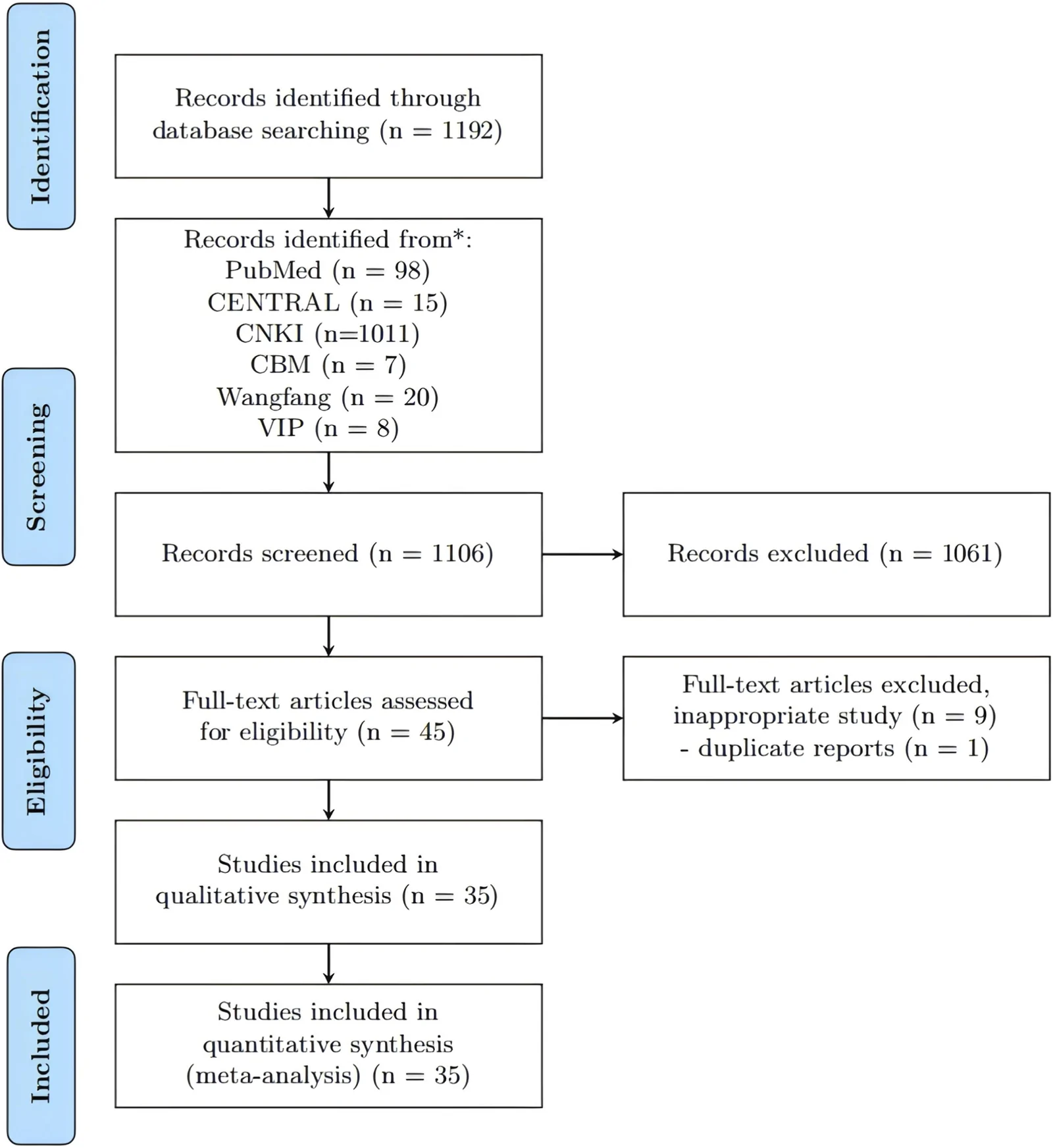
Flow chart for screened articles.

### 3.2 Index case: dasatinib-induced chylothorax

A 14-year-old boy with Ph + ALL had been receiving dasatinib 100 mg/day alongside intensive chemotherapy according to the ALL-I/HR protocol. He continued on dasatinib for 30 months and remained in remission, with minimal residual disease consistently below 0.01% and *BCR::ABL* P190 negative. On 11 December 2025, a routine chest computed tomography (CT) scan incidentally revealed bilateral pleural effusion. Thoracentesis produced milky-yellow fluid, and biochemistry left no doubt about chylothorax: the pleural fluid triglyceride concentration came back at 20.40 mmol/L (roughly 1806 mg/dL), cholesterol was 4.08 mmol/L, and lymphocytes made up about 90% of the cells. Bone marrow examination ruled out leukaemic relapse, and there was no history of thoracic trauma, surgery, or mediastinal mass to explain the effusion. A fuller set of laboratory results can be found in Tables 1–4.

**TABLE 1:** Clinical timeline of the index patient.

Time point	Core clinical events
31 May 2023	Diagnosed with Ph + Early Pre B-ALL; induction chemotherapy launched
05 June 2023	Initiated oral dasatinib 100 mg qd; sustained complete bone marrow remission post chemotherapy
10 December 2025	Completion of all chemotherapy cycles; continuous dasatinib administration for 2 years and 6 months
Day 1 (11 December 2025)	New-onset chest tightness and fatigue; chest CT showed moderate bilateral pleural effusion, hospital admission
Day 2 (13 December 2025)	Thoracentesis verified chylothorax; pleural fluid mNGS positive for Bartonella henselae; ceftazidime and prophylactic sulfamethoxazole-trimethoprim given
Day 5 (15 December 2025)	Permanent discontinuation of dasatinib; antibiotic switched to cefoperazone-sulbactam; doxycycline + rifampin started for anti-Bartonella treatment. Bone marrow and lumbar puncture excluded leukemia relapse and CNS infection
Day 16 (26 December 2025)	Follow-up CT demonstrated significant absorption of pleural effusion; repeated pleural fluid mNGS only detected human herpesvirus 6
Day 17 (27 December 2025)	Complete resolution of respiratory symptoms; intravenous therapy stopped, patient discharged on oral drugs for follow-up

Chronological clinical events of the 14-year-old male patient with Philadelphia-positive early precursor B-cell acute lymphoblastic leukemia (Ph + Early Pre B-ALL), dasatinib-associated chylothorax, and concurrent *Bartonella henselae* infection. ALL, acute lymphoblastic leukemia; CT, computed tomography; mNGS, metagenomic next-generation sequencing.

**TABLE 2:** Laboratory findings in blood before and after treatment.

Blood	Result (Day 3)	Result (Day 16)
White blood cells	2.5 × 10^9^/L	3.3 × 10^9^/L
Neutrophil count	1.60 × 10^9^/L	1.92 × 10^9^/L
Serum total protein	59.6 g/L	46.3 g/L
Serum lactate dehydrogenase	293 U/L	162 U/L
Haemoglobin	170 g/L	129 g/L
Platelets	258 × 10^9^/L	283 × 10^9^/L
Creatinine	46 μmol/L	52 μmol/L
Glucose	6.41 mmol/L	7.12 mmol/L
Total cholesterol	3.77 mmol/L	3.79 mmol/L
Triglycerides	0.67 mmol/L	1.43 mmol/L

**TABLE 3:** Laboratory findings in chylothorax before and after treatment.

Pleural fluid	Result (Day 3)	Result (Day 16)
Gross appearance	milk yellow and turbid	yellow and clear
Qualitative Protein Test	Positive++	Positive
Nucleated Cell	1,265 × 10^6^/L	2,266 × 10^6^/L
segmented neutrophil	—	1.00%
Monocytes	10%	—
Lymphocytes	90.00%	98.00%
Reactive lymphocytes	—	1.00%
Red blood cell count	7,000 × 10^6^/L	1,000 × 10^6^/L
Erythrocyte morphology	Fresh erythrocytes	Fresh erythrocytes
Hemosiderin-laden alveolar macrophages	Not detected	Not detected
Chylous Test	Positive	Positive
Glucose	6.05 mmol/L	2.98 mmol/L
Lactate dehydrogenase	133.0 U/L	215.0 U/L
Total protein	43.1 g/L	23.7 g/L
Pleural fluid adenosine deaminase	6.9 U/L	9.8 U/L
Metagenomic next-generation sequencing	*Bartonella henselae*, Reads 66, Confidence medium; *Enterobacter cloacae* complex, Reads 62, Confidence medium; *Acinetobacter baumannii*, Reads 14, Confidence medium; Human respiratory syncytial virus A, Reads 96, Confidence medium	Human herpesvirus 6, reads 127, confidence medium
Pleural fluid culture	Negative	—
X-PERT	Negative	—
Cytopathology	Mesothelial cells and lymphocytes were observed, no malignant cells seen	—
Homocysteine	<3.00 μmol/L	—
Pleural fluid total cholesterol	4.08 mmol/L	—
Pleural fluid triglycerides	20.40 mmol/L	—
High-density lipoprotein cholesterol	0.27 mmol/L	—
Low-density lipoprotein cholesterol	0.4 mmol/L	—
Apolipoprotein A1	0.82 g/L	—
Apolipoprotein B	<0.15 g/L	—

**TABLE 4:** Computed tomography of the chest before and after treatment.

Computed tomography (Figure 3)	(Day 1) Moderate bilateral pleural effusion with subpleural atelectasis in the bilateral lower lung lobes	(Day 16) Bilateral indwelling chest tubes are observed, with trace bilateral pleural effusion. Compared with the prior CT examination dated 11 December 2025, there is a significant reduction in the bilateral pleural effusion, accompanied by re-expansion of the subpleural lung parenchyma in both lower lobes. The inflammatory lesion in the left upper lobe has completely resolved. A nodule in the right lower lobe is now more clearly defined than on the previous study

Applying the Naranjo Adverse Drug Reaction Probability Scale to the index case (Table 5) yields a score of 7 (“probable”): previous reports of dasatinib-induced chylothorax (+1), temporal relationship (+2), absence of alternative aetiologies (+2), objective confirmation by pleural fluid TG (+1), and improvement after drug discontinuation (+1) (Naranjo et al., 1981). Dasatinib was discontinued straight away. Bilateral chest drains were placed, the patient was kept fasting on total parenteral nutrition, and subcutaneous somatostatin was started. Over time, chest tube output fell from more than 1,000 mL per 24 h to under 300 mL, and the drainage fluid gradually turned from milky-yellow to clear (Figure 2). Two weeks later, a follow-up chest CT showed near-complete resolution of the pleural effusion and good re-expansion of the lungs (Figure 3). Dasatinib was permanently stopped, and no recurrence of the effusion was seen during follow-up.

**TABLE 5:** Naranjo Adverse Drug Reaction Probability Scale assessment indicating dasatinib as the probable cause of chylothorax in the index case.

Parameter	Score	Rationale
Previous conclusive reports of the reaction	+1	Dasatinib-induced chylothorax has been well documented in published case reports
Plausible temporal relationship	+2	Chylothorax developed after 30 months of continuous dasatinib treatment
No alternative etiologies identified	+2	No trauma, surgery, malignancy, or other infectious causes were detected to explain chylothorax
Objective laboratory confirmation	+1	Diagnosis confirmed by extremely high pleural fluid triglyceride (20.40 mmol/L)
Clinical improvement after drug cessation	+1	Pleural effusion resolved rapidly after dasatinib discontinuation
Total score	7	Category: probable adverse drug reaction

**FIGURE 2:**
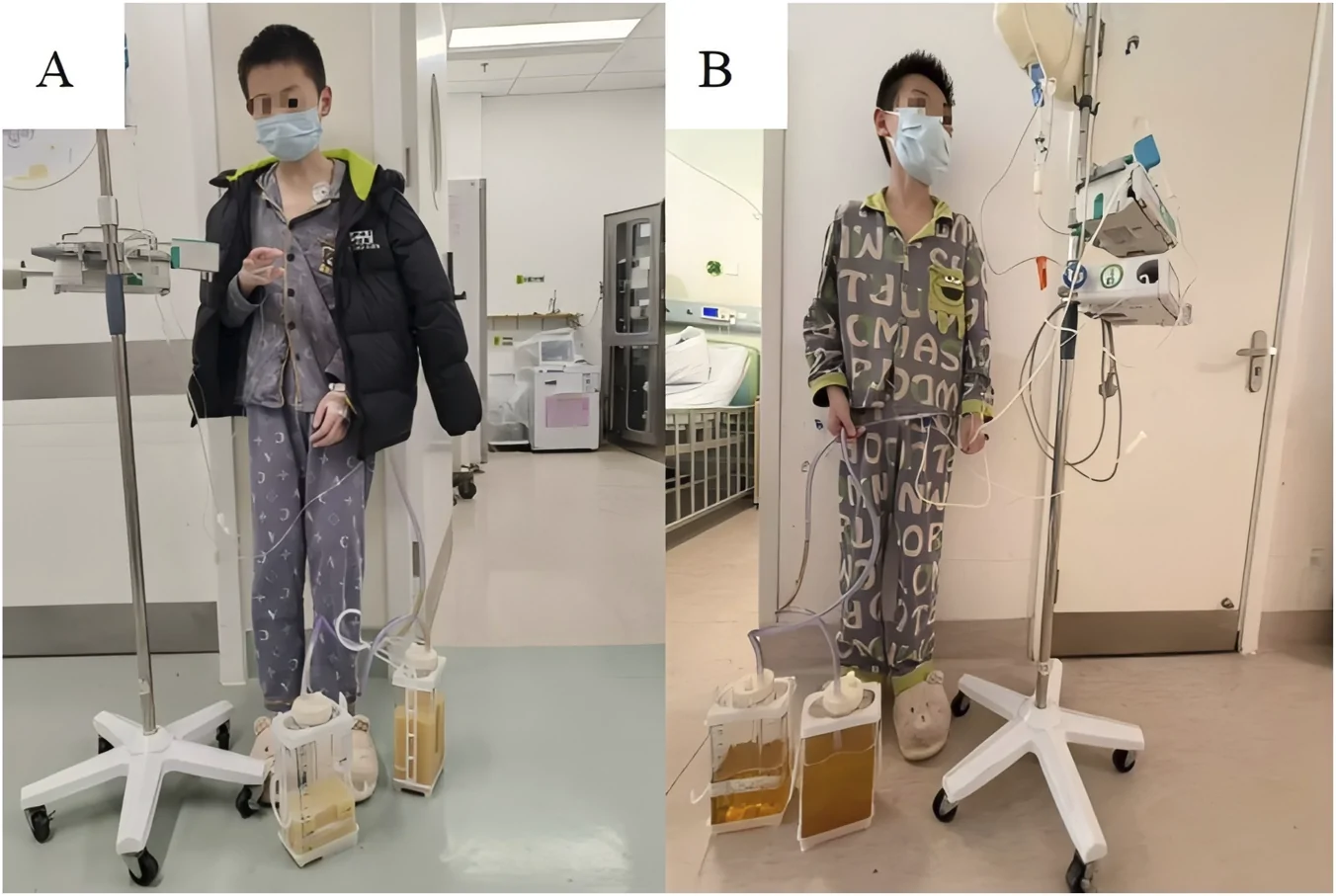
Macroscopic appearance of the pleural fluid **(A)** Milky turbid chylous fluid obtained at initial drainage before treatment. **(B)** Clear yellow drainage fluid after 2 weeks of treatment.

**FIGURE 3:**
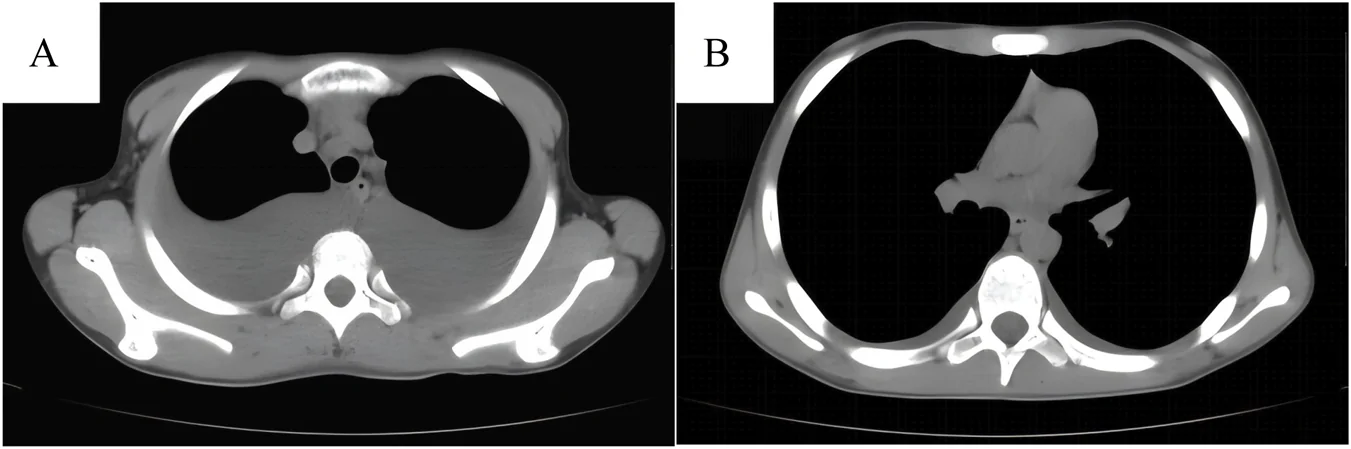
Corresponding computed tomography (CT) findings before and after treatment for chylothorax. **(A)** Chest CT with mediastinal window showing bilateral chylothorax before treatment (Day 1). **(B)** Follow-up chest CT with mediastinal window showing near-complete resolution of pleural effusion and lung re-expansion (Day 16).

### 3.3 Characteristics of TKI-associated chylothorax cases (including index case)

Twenty-seven cases were analysed (Table 6), including three cases from one published report and our index patient. The median age was 52 years (range 5–79 years), with only five paediatric patients (≤18 years). Most patients had CML (n = 22, 84%), four had Ph+ ALL, and one had non-small cell lung cancer treated with alectinib. Dasatinib was the causative agent in 26 of 27 TKI-related cases. The median time to chylothorax onset was 22.5 months (range 8 days–14 years). The median pleural fluid TG concentration was 506 mg/dL (range 109–1806 mg/dL), with our index case recording the highest value.

**TABLE 6:** The information on cases of chylothorax caused by TKIs.

Case	Country	Age (years)	Gender	Diagnosis	Duration of TKIs	Previous treatment	Dose	Discontinuation	Recurrence	Appearance	TG (mg/dL)	Laterality	Treatment for chylothorax	Ref.
1	China	40	Female	CML	40 months	Imatinib	50 mg twice a day	Yes, switched to nilotinib	Yes	milky white	right: 263; left: 536	Bilateral	Thoracentesis, diuretics, steroids	(Al-Abcha et al., 2019)
2	Kuwait	18	Female	CML	63 months	Imatinib	100 mg daily	Yes, switched to nilotinib	No	milky white	584.1	Bilateral	Thoracentesis	(Alqattan et al., 2022)
3	Spain	53	Male	CML	14 years	NR	NR	Yes	No	milky white	582.33	Left	Thoracentesis, octreotide	(Bajaj et al., 2023)
4	Japan	73	Female	CML	1 year	Dasatinib	70 mg daily	Yes, switched to bosutinib then imatinib	Yes	milky white	273	Right	Thoracentesis, diuretics, Japanese herb “Goreisan”	(Baloch et al., 2017)
5	China	71	Male	CML	6 months	Imatinib	100 mg daily	Yes	Yes	milky white	226.56	Bilateral	Thoracentesis	(Bradt et al., 2022)
6	United States	63	Female	CML	NR	NR	80 mg daily	Yes, switched to nilotinib	No	NR	700	Bilateral	Thoracentesis	(Chen et al., 2020)
7	United States	79	Female	CML	1 year	NR	NR	Yes, switched to bosutinib	No	milky white to pink	139	Bilateral	Thoracentesis	(Ferreiro et al., 2016)
8	Singapore	63	Male	CML	45 months	NR	NR	Yes, switched to imatinib	NR	milky white to pink	620	Left	Thoracentesis	(Ga et al., 2023)
9	Spain	63	Female	CML	1 year	NR	100 mg daily	Yes, switched to imatinib	No	milky white	334	Left	Thoracentesis, steroids	(Hickman et al., 2020)
10	China	43	Male	CML	4 years	Dasatinib	100 mg daily	Yes, switched to nilotinib	No	yellow	728	Bilateral	Thoracentesis, steroids, diuretics	(Hsu et al., 2021)
11	Belgium	67	Male	CML	8 months	Dasatinib	100 mg daily	Yes, switched to bosutinib	NR	red	969	Bilateral	Thoracentesis, diuretics	(Huang et al., 2015)
12	United States	39	Female	CML	10 years	Dasatinib	50 mg daily	Yes	No	NR	481	Bilateral	Thoracentesis, diuretics, steroids	(Justin et al., 2025)
13	United States	5	Female	CML	2 years	Imatinib	150 mg/m^2^	Yes	No	milky white	603	Bilateral	Thoracentesis	(Kelly et al., 2022)
14	Spain	72	Female	CML	10 years	Imatinib, nilotinib	100 mg daily	Yes	NR	milky white to pink	251	Right	Thoracentesis, diuretics, steroids	(Liu et al., 2023)
15	United States	69	Male	CML	10 months	Imatinib, nilotinib	100 mg daily	No, reduced to 50 mg qd	Yes	milky white	405	Right	Thoracentesis	(Makimoto et al., 2022)
16	China	11	Male	CML	38 months	Imatinib	50 mg twice a day	Yes	No	milky white to pink	NR	Bilateral	Thoracentesis, fasting, diuretics, steroids	(Martínez et al., 2023)
17	United States	74	Male	CML	NR	Dasatinib	80 mg daily	Yes, switched to imatinib	Yes	milky white to pink	492	Right	Thoracentesis	(McGonegal and Baskar, 2024)
18	Singapore	12	Male	Ph-positive ALL	18 months	Dasatinib	90 mg daily	Yes	NR	milky white	109.74	Right	Thoracentesis, diuretics, steroids	(Molina et al., 2020)
19	Japan	43	Male	CML	10 years	Imatinib, nilotinib	100 mg daily	Yes, switched to bosutinib	No	milky white to pink	1,374	Left	Thoracentesis	(Nasi et al., 2022)
20	Spain	71	Female	Ph-positive ALL	2 months	Imatinib	140 mg daily	NR	NR	milky white to pink	right: 625; left: 378	Bilateral	Thoracentesis	(Ng et al., 2024)
21	China	51	Male	CML	50 months	Imatinib	50 mg twice a day	Yes, switched to nilotinib	No	yellow	135	Bilateral	Thoracentesis	(Pai and Huang, 2025)
22	China	47	Male	CML	8 months	Imatinib	100 mg daily	No	No	milky white	NR	Right	Thoracentesis, diuretics	(Paul et al., 2021)
	China	46	Male	CML	19 months	Imatinib	100 mg daily	Yes, switched to imatinib	No	milky white	NR	Left	Thoracentesis, diuretics	
	China	49	Male	CML	26 months	Imatinib	100 mg daily	Yes	No	milky white	1,054	Bilateral	Thoracentesis, fasting	
23	Katar	51	Male	Ph-positive ALL	20 months	Dasatinib	140 mg daily	Yes	No	milky white	1,345.2	Bilateral	Thoracentesis, diuretics, steroids	(Sasaki et al., 2019)
24	United States	30	Male	NSCLC	8 days	Alectinib	600 mg twice a day	Yes, switched to Brigatinib	Yes	milky white	975	Bilateral	Thoracentesis	(Yang et al., 2016)
25	China	14	Male	Ph-positive ALL	30 months	Dasatinib	100 mg daily	Yes	No	milky yellow	1806	Bilateral	Thoracentesis, fasting, diuretics, somatostatin	our case

CML, chronic myeloid leukemia; Ph-positive ALL, Philadelphia chromosome-positive acute lymphoblastic leukemia; NSCLC, Non-Small Cell Lung Cancer.

All patients discontinued or reduced the TKI dose; 24 of 27 permanently discontinued dasatinib. Therapeutic thoracentesis was universal, with adjunctive measures including diuretics, corticosteroids, somatostatin, and fasting. The most common alternative TKIs were nilotinib (n = 9), bosutinib (n = 5), and imatinib (n = 4). Chylothorax recurred in six patients, including some after switching to alternative TKIs, suggesting a potential class effect.

### 3.4 Index case: concurrent *Bartonella henselae* infection

Metagenomic next-generation sequencing (mNGS) of the initial pleural fluid detected *B. henselae* (66 reads, medium confidence), together with *Enterobacter cloacae* complex, *Acinetobacter baumannii*, and respiratory syncytial virus A. Notably, no serological or polymerase chain reaction (PCR) testing was undertaken for Bartonella; the diagnosis of cat-scratch disease was ultimately established by integrating this single mNGS finding with the patient’s history of indirect contact with stray cats (without a documented scratch or bite) and the physical examination finding of mild left conjunctival injection. He was consequently treated with doxycycline 100 mg twice daily plus rifampicin 600 mg once daily for 2 weeks. A repeat mNGS assay subsequently confirmed clearance of *B. henselae*.

### 3.5 Characteristics of *Bartonella henselae* infection cases in leukemia (including index case)

Twelve cases (including our index patient) were identified (Table 7). The most common underlying disease was chronic lymphocytic leukemia (CLL, n = 8), followed by ALL (n = 2), CML (n = 1), and acute myeloid leukemia (n = 1). Only four patients had a clear history of cat scratch; eight had only indirect feline contact. Clinical manifestations ranged from localised lymphadenopathy to visceral involvement (neuroretinitis, osteomyelitis). Our case was unique in that *B. henselae* was detected only in pleural fluid, with mild conjunctival injection as the sole clinical sign.

**TABLE 7:** The information on cases of leukemia combined with cat-scratch disease.

Case	Country	Age (years)	Gender	Diagnosis	Chemotherapeutics	Transplant	Clinical manifestations	Infection history	Cell count	Laboratory findings	Treatment	Outcome	References
1	United States	6	Female	T-ALL	Nelarabine, methotrexate, vincristine, mercaptopurine, prednisone	NR	Lymphadenopathy and frontal lobe lesions	Only cat contact, no scratch history	WBC: 3.5 × 10^9^/L, NEUT normal	Bartonella serology negative, PCR positive	Doxycycline and sulfonamides	Resolution of enhancing brain lesions and intra-abdominal lymphadenopathy	(Sadanand et al., 2024)
2	United States	52	Male	CLL	NR	NR	Right axillary mass, night sweats, weight loss, low-grade intermittent fever for 1 week	Only cat contact, no scratch history	WBC: 21 × 10^9^/L, lymphocytes: 7.7 × 10^9^/L	*B. henselae* IgG and IgM positive, titers >1:256	Azithromycin for 2 months	Rash resolved, right arm abscess resolved, lymph nodes remained enlarged	(Razaq et al., 2005)
3	United States	65	Female	AML	NR	NR	Knee joint redness, swelling, tenderness, low-grade fever	Yes	Synovial fluid WBC: 1,295/μL	CRP: 87.2 mg/L, ESR: 94 mm/h, Bartonella PCR positive	Doxycycline 42 days and rifampin 16 days	Knee pain and swelling improved	(Haimes and Nelms, 2019)
4	United States	15	Female	CML	Imatinib	Yes	Right eye vision loss, right neck swelling with intermittent headache	No	NR	Initial *B. henselae* IgG titer 1:128, repeat IgG titer 1:512 after 7 days	Doxycycline and rifampin for 2 weeks	Vision recovered, macular edema resolved, submandibular lymph nodes reduced	(Irshad and Gordon, 2009)
5	United States	76	Male	CLL	NR	NR	Tender lymphadenopathy in right axilla	Yes	WBC: 10.2 × 10^9^/L, lymphocytes 42%	Blood culture and Bartonella serology negative	Azithromycin for 3 weeks	Axillary lymph nodes spontaneously ruptured with purulent discharge and symptom relief	(Balakumar et al., 2019)
6	United States	23	Male	T-CLL	Methotrexate, vincristine, mercaptopurine, prednisone	NR	Right inguinal lymphadenopathy	Only cat contact, no scratch history	WBC: 3.6 × 10^9^/L, neutrophils 80%	IgM negative, IgG >1:1,024	Azithromycin for 10 days	Lymph node regression	(Jeong et al., 2005)
7	China	31	Female	CLL	Zanubrutinib	NR	Persistent high fever, bilateral tonsillar enlargement, multiple painless lymphadenopathy in neck, right axilla, and inguinal regions	Only cat contact, no scratch history	WBC: 11.98 × 10^9^/L, NEUT: 6.92 × 10^9^/L, lymphocytes: 4.28 × 10^9^/L	hs-CRP: 56.94 mg/L, mNGS: *B. henselae* sequences 38, relative abundance 71.765%	Moxifloxacin, gentamicin sulfate, azithromycin, and doxycycline for 6 days; oral therapy for 1 month	Body temperature normalized, lymphadenopathy reduced	(Luan et al., 2026)
8	Germany	55	Male	B-CLL	Chlorambucil, cyclophosphamide, vincristine, prednisone	NR	Multiple hemangioma-like skin lesions throughout body	No	WBC: 4.4 × 10^9^/L, neutrophils: 0.73 × 10^9^/L, lymphocytes: 0.11 × 10^9^/L	GMS stain positive for argyrophilic bacteria, EM confirmed Bartonella	Clarithromycin for 4 weeks	Fever resolved, skin papules resolved, subcutaneous abscess healed	(Milde et al., 1995)
9	Australia	66	Female	CLL	Fludarabine, cyclophosphamide, rituximab	NR	Hemangioma-like skin lesions on face, scalp, arms, and trunk; progressed to left foot osteomyelitis after 12 weeks	No	WBC: 5.8 × 10^9^/L, neutrophils: 0.52 × 10^9^/L	ESR: 42 mm/h, CRP: 37.5 mg/L, skin biopsy Bartonella PCR negative, IgG <1:128	Doxycycline + clarithromycin 12 weeks (ineffective), gentamicin 6 weeks followed by oral azithromycin + rifampin 15 months	Left foot swelling resolved after 6 weeks, skin lesions slowly improved after 15 months	(Holmes et al., 2011)
10	Hungary	78	Male	CLL	Lomefloxacin, prednisone	NR	Papular skin lesions on face and arms	Yes	WBC: 27 × 10^9^/L, neutrophils 9%	ESR: 33 mm/h, GMS stain positive for argyrophilic bacteria, EM conffrmed Bartonella	Erythromycin for 6 weeks	Hemangioma-like rash completely resolved after 8 weeks	(Török et al., 1994)
11	United States	60	Male	CLL	Cyclophosphamide, doxorubicin, vincristine, prednisone	NR	Fever, skin nodules on right forearm	Yes	WBC: 32.5 × 10^9^/L, neutrophils: 0.65 × 10^9^/L, lymphocytes: 0.11 × 10^9^/L	Serum Bartonella IgG and IgM IFA negative	Doxycycline for 6 weeks	Nodules resolved after 6 weeks	(Petersen et al., 2008)
12	China	14	Male	ALL	Vincristine, daunorubicin, pegaspargase, dexamethasone	No	Mild conjunctival hyperemia of left eye, otherwise unremarkabl	Only cat contact, no scratch history	WBC: 3.5 × 10^9^/L, NEUT: 2.84 × 10^9^/L	Pleural effusion mNGS detected *B. henselae*	Doxycycline and rifampin for 2 weeks	*B. henselae* cleared from pleural effusion mNGS after 2 weeks	Our case

T-ALL, T-Cell Acute Lymphoblastic leukemia; CML, Chronic Myeloid leukemia; CLL, Chronic Lymphocytic leukemia; AML, Acute Myeloid leukemia; T-CLL, T-Cell Chronic Lymphocytic leukemia; B-CLL, B-Cell Chronic Lymphocytic leukemia; ALL, Acute Lymphoblastic leukemia; WBC, White Blood Cell Count; CRP, C-Reactive Protein; ESR, Erythrocyte Sedimentation Rate; PCR, Polymerase Chain Reaction; GMS, Grocott’s Methenamine Silver Stain; EM, Electron Microscopy; mNGS, Metagenomic Next-Generation Sequencing.

Diagnosis was made by serology (n = 5), PCR/mNGS (n = 5), or histopathology (n = 2). Three immunocompromised patients had negative serology. All patients received antibiotic therapy, most commonly doxycycline plus rifampicin or a macrolide, and 11 of 12 achieved clinical resolution.

## 4 Discussion

This study summarizes currently available evidence regarding dasatinib-associated chylothorax and *B. henselae* infection in leukemia patients and documents, for the first time, the coexistence of both conditions in a single patient.

The pleural fluid triglyceride concentration observed in our patient represents the highest level reported to date. This finding supports the presence of severe lymphatic endothelial dysfunction associated with prolonged dasatinib exposure. Previous studies have suggested that inhibition of PDGFR-β signaling disrupts pericyte recruitment and lymphatic vessel integrity, whereas Src-family kinase inhibition impairs endothelial barrier function and vascular permeability.

Importantly, Src-family kinases also play a central role in T-cell receptor signaling and adaptive immune activation. Dasatinib-mediated suppression of T-cell immune surveillance may therefore contribute to increased susceptibility to opportunistic infection. It is conceivable that the lipid-rich chylous pleural environment, together with localized immune dysfunction, might create a permissive microenvironment that could favor *B. henselae* persistence. However, this remains a hypothesis that requires further validation.

The present findings additionally underscore the potential diagnostic value of metagenomic next-generation sequencing in immunocompromised patients with unexplained pleural effusion. In several published cases, conventional serological assays were negative despite confirmed Bartonella infection. Early molecular diagnostic testing may therefore facilitate timely identification of occult opportunistic pathogens.

Management of dasatinib-associated chylothorax generally requires prompt drug discontinuation, supportive drainage procedures, nutritional modification, and consideration of alternative TKIs. Published evidence suggests that bosutinib may be associated with lower recurrence risk compared with dasatinib rechallenge, although long-term pediatric safety data remain limited.

### 4.1 Mechanisms of dasatinib-induced chylothorax and immunological implications

Dasatinib exhibits low kinase selectivity, inhibiting BCR-ABL, c-KIT, EphA2, PDGFR-β, and Src family kinases (including Fyn, Yes, Src, and Lyn). Several mechanistic pathways have been proposed to explain its lymphatic adverse effects. PDGFR-β inhibition disrupts pericyte recruitment and mural cell function, weakening lymphatic vessel walls (Bergers et al., 2003; Cao et al., 2004). Simultaneously, dasatinib blocks Src family kinases, which phosphorylate vascular endothelial-cadherin and regulate endothelial barrier integrity; Src inhibition reduces VEGF-dependent signalling and increases endothelial permeability (Phan et al., 2018; Wallez et al., 2007). Preclinical studies have confirmed that dasatinib generates reactive oxygen species in pulmonary endothelial cells, enhancing permeability and promoting pleural effusion formation (Phan et al., 2018).

Critically, Src family kinases also play a pivotal role in T-cell receptor (TCR) signal transduction. Lck, a Src family kinase, is essential for TCR-mediated activation, and its inhibition impairs adaptive immune responses (Palacios and Weiss, 2004). Thus, dasatinib may concurrently inhibit Src kinases in both lymphatic endothelium and T lymphocytes. The endothelial damage could lead to chylous leakage, while the immunological impairment might weaken cellular immune surveillance. The accumulating lipid-rich chylous effusion may provide a nutrient-replete environment, and the compromised cellular immunity could potentially facilitate the survival of intracellular pathogens such as *B. henselae*. The observation that *B. henselae* was detected only in the chylous pleural fluid, without systemic signs of infection, could be interpreted as suggestive of a localized immune-privileged niche within the pleural space. This hypothesis offers one possible explanation for the concurrent occurrence of these two rare conditions, but it remains speculative.

To date, no published study has specifically investigated the association between *Bartonella henselae* infection and chylothorax. In the context of our index case, several interpretations are possible. First, the detection of *B. henselae* in pleural fluid might be incidental, representing a coincidental finding without any pathogenic contribution to the effusion. Second, dasatinib-induced lymphatic injury might have facilitated the localization and persistence of *B. henselae* in the pleural space, and the organism could have contributed to or exacerbated the effusion through local inflammatory effects. Third, there might be no causal relationship between the two conditions; their coexistence could be purely fortuitous. Given the limited evidence from a single case, we cannot distinguish among these possibilities, and further research is needed to explore any potential mechanistic interplay.

### 4.2 Paediatric considerations and TKI selection

We know very little about dasatinib-associated chylothorax in children—only five cases, including the one we report here, have been documented in patients aged 18 years or younger. Pharmacokinetic data in paediatric populations are scarce, but what there is suggests that drug exposure can vary quite a bit depending on whether dosing is calculated by body surface area or given as a flat dose, and that variability could contribute to unpredictable lymphatic toxicity. Our patient received 100 mg daily, which works out to roughly 70 mg/m^2^ and falls within the usual therapeutic range, yet he went on to develop the highest pleural fluid TG level reported to date. That sort of extreme value points to individual susceptibility factors that go beyond dosing alone.

Once dasatinib is stopped, picking the right alternative TKI becomes a key decision. Looking across the published cases in Table 6, the two most common switches were to nilotinib and bosutinib. The NCCN Clinical Practice Guidelines in Oncology: Chronic Myeloid leukemia (Version 1.2025) indicate that dasatinib is associated with a higher incidence of pleural effusion recurrence than bosutinib (Shah et al., 2024). That said, some second-generation TKIs come with their own cardiac and vascular baggage, so the risk–benefit balance has to be worked out for each patient individually. Our patient stayed in sustained remission after finishing his planned treatment and did not need another TKI, but for those who do require ongoing therapy, bosutinib looks like the safer bet when it comes to avoiding recurrent chylothorax.

### 4.3 *Bartonella henselae* infection in immunocompromised hosts

Patients with leukemia are already more vulnerable to infection because of the immunosuppression that comes from both the disease itself and its treatment, although *B. henselae* infection remains relatively uncommon even in this population. What our review brings home is that a missing scratch history does not rule anything out—eight of twelve cases had no documented scratch or bite—and in the immunocompromised setting, serology can be falsely negative, as it was in three cases. Our case stands apart because *B. henselae* was found only in the pleural fluid by mNGS, with mild conjunctival injection as the sole clinical clue. Without that comprehensive molecular diagnostic work-up, it is almost certain the infection would have been missed. This fits with the idea that the dasatinib-treated pleural space can function as a localised immune-privileged niche, letting the pathogen settle in without any systemic spread. mNGS, with its unbiased and high-sensitivity detection capability, proved instrumental in establishing the diagnosis in our case and should be considered early in leukemia patients with unexplained pleural effusion, particularly when conventional diagnostic tests are negative (Jacob et al., 2019). Once identified, targeted therapy with doxycycline and rifampicin is effective, and treatment duration should be individualised based on clinical response (Angelakis and Raoult, 2014) and, when available, molecular clearance as confirmed by repeat mNGS.

### 4.4 Limitations

This review has all the usual limitations of a case-report-based analysis—publication bias and patchy data reporting. In quite a few cases, details on immunosuppressive regimens or haematopoietic stem cell transplantation status were missing, which meant we could not properly gauge how much those factors might have influenced infection susceptibility. The mechanistic ideas put forward about how chylothorax develops are biologically plausible, but they still sit at the hypothesis level and need experimental validation. Finally, long-term follow-up data on the safety of alternative TKIs in children are almost nonexistent, so conclusions on that front have to be drawn with real caution.

## 5 Conclusion

Dasatinib-associated chylothorax is rare, but when it occurs it can be severe—and it can show up even after a long period on treatment. Our index case suggests that an occult *B. henselae* infection can coexist with chylothorax, probably helped along by dasatinib’s dual hit on lymphatic integrity and T-cell immunity. For clinicians looking after paediatric leukemia patients on long-term TKIs, the practical message is straightforward: stay alert for both lymphatic complications and atypical opportunistic infections. The extremely high pleural fluid TG level in this case drives home just how profound the lymphatic injury can be. Getting a good outcome depends on stopping the drug promptly, switching to an alternative TKI when needed, providing supportive care, and using molecular diagnostic tools like mNGS. Going forward, we need studies that dig into the molecular basis of dasatinib-induced lymphatic damage and that compare the safety profiles of alternative TKIs in children more systematically.

## Data Availability

The original contributions presented in the study are included in the article/supplementary material, further inquiries can be directed to the corresponding authors.
